# Unveiling the Brain-Penetrating Material Basis of Dragon’s Blood: Identification of Active Metabolites and Metabolic Pathways for Ischemic Stroke Therapy

**DOI:** 10.3390/metabo16050327

**Published:** 2026-05-14

**Authors:** Yu Zhu, Jiahui Ren, Meijia Chen, Jianglong Chen, Guang Li

**Affiliations:** 1 Institute of Medicinal Plant Development, Chinese Academy of Medical Sciences & Peking Union Medical College, Beijing 100193, China; s2023009012@student.pumc.edu.cn (Y.Z.);; 2 Yunnan Branch, Institute of Medicinal Plant Development, Chinese Academy of Medical Sciences & Peking Union Medical College, Xishuangbanna 666100, China

**Keywords:** dragon’s blood, metabolites, Borneol, UHPLC-Q-TOF-MS/MS, cerebrospinal fluid

## Abstract

**Background:** Dragon’s blood (dried resin of *Dracaena cochinchinensis (Lour.) S.C.Chen*) is a classic traditional medicine for treating ischemic stroke, yet its bioactive components capable of penetrating the blood–brain barrier (BBB) remain ill-defined. This study aims to elucidate its material basis and the synergistic mechanism of Borneol as a “guide drug.” **Methods:** A systematic strategy integrating UHPLC-Q-TOF-MS/MS and metabolomics was employed to map the chemical profile of dragon’s blood and identify its migrating constituents in rats. **Results:** A total of 96 compounds were characterized in vitro. In vivo analysis of the cerebrospinal fluid (CSF) revealed a brain-penetrating profile that was significantly enriched by Borneol, with the number of detected constituents increasing from 11 in the DB group to 16 in the DB + B group. The results demonstrated that demethylation, glycoside hydrolysis, and oxidation are primary metabolic pathways, validating a “pro-drug” mechanism where aglycones and hydroxylated derivatives act as the central effectors. Notably, Borneol not only enhanced the BBB permeability of lipophilic flavonoids but also facilitated unique metabolic transformations, such as the cyclization of berberrubine to coptisine. **Conclusions:** This study elucidates the brain-penetrating material basis of dragon’s blood and reveals the dual synergistic mechanism of Borneol involving both physical permeation enhancement and metabolic modulation, offering scientific evidence for its clinical application in central nervous system diseases.

## 1. Introduction

*Dracaena cochinchinensis (Lour.) S.C.Chen*, a highly valued species within the Asparagaceae family, represents a cornerstone of traditional ethnomedicine in South and Southeast Asia. The deep-red resin extracted from its stems, commonly known as dragon’s blood (referred to as “Xueshie” in Chinese), is a classic botanical drug with profound phytochemical diversity and pharmacological potential. Geographically, it is predominantly distributed across the tropical and subtropical regions of Southeast Asia, including Vietnam, Laos, Cambodia, and the southern border provinces of China (e.g., Yunnan and Guangxi). For centuries, indigenous communities have utilized this resinous plant part to activate blood circulation, dissipate blood stasis, and relieve pain, making it highly relevant for treating cardiovascular and cerebrovascular diseases such as ischemic stroke. To systematically align with the research theme of uncovering the therapeutic promise of Southeast Asia’s medicinal plants, the geographic distribution, local name and ethnomedicinal applications of *D. cochinchinensis* are summarized in Table 1.

Cerebral ischemia, a common clinical cerebrovascular disease, is characterized by high incidence, a high disability rate, and a high recurrence rate. Its pathogenesis is closely associated with complex pathological processes such as oxidative stress, inflammatory response, and neuronal apoptosis triggered by cerebral tissue ischemia and hypoxia [7]. Current clinical treatments primarily focus on improving blood flow perfusion, making it challenging to simultaneously achieve neuroprotection and reverse pathological damage. Traditional Chinese medicine (TCM), however, demonstrates unique potential in treating cerebral ischemia due to its multi-component, multi-targeted therapeutic advantages. dragon’s blood Resin, a classic Chinese medicine for promoting blood circulation and removing blood stasis, is derived from the resin of *Dracaena cochinchinensis (Lour.) S.C.Chen*. Modern pharmacological studies confirm that its flavonoids, terpenoids, phenolic acids, and other components exhibit activities such as anti-cerebral ischemia, inflammation inhibition, and reduction in blood–brain barrier damage [8]. In our previous study [9], we demonstrated the neuroprotective effects of dragon’s blood in transient middle cerebral artery occlusion (tMCAO) model rats. However, the core material basis for its pharmacological effects—namely, the active components that enter the bloodstream after oral administration and reach brain tissue, along with their in vivo transformation patterns—remains incompletely elucidated.

The in vivo processes of traditional Chinese medicine (absorption, distribution, metabolism, excretion) directly determine its therapeutic efficacy. Only components that enter the bloodstream and their metabolites can potentially cross the blood–brain barrier to act on diseased brain tissue. Therefore, deciphering the blood-entering prototypical components of dragon’s blood resin and its in vivo transformation pathways is crucial for elucidating the material basis of its treatment for cerebral ischemia. Previous studies on the in vivo components of traditional Chinese medicines have established a mature technical paradigm [10]. For instance, two-dimensional ultra-high-performance liquid chromatography coupled with quadrupole-orbitrap high-resolution mass spectrometry (2D-UHPLC-Q-Orbitrap HRMS) enables efficient separation and structural characterization of trace components in complex systems. Combining UHPLC-Q-TOF-MS/MS with bioinformatics tools (such as Compound Discoverer and MS-DIAL software) enables precise identification of bioavailable prototypes and metabolites in biological samples. Simultaneously, comparing biological samples (plasma, brain tissue, etc.) from the administered group with those from the control group eliminates endogenous interference, clarifying the in vivo fate of unique TCM components. These technical approaches provide important references for studying the bioavailable components and in vivo transformation of dragon’s blood resin.

To further enhance the central delivery of these therapeutics, Borneol is classically co-administered as a “guide drug” (Yin-Jing drug) in TCM formulations. Despite its widespread clinical use, the precise synergistic mechanism by which Borneol facilitates the CNS exposure of dragon’s blood constituents—whether through physical permeation enhancement or metabolic modulation—requires rigorous investigation.

This study proposes to leverage the aforementioned established technological pathways to construct a systematic research framework encompassing an “in vitro component characterization—in vivo blood entry identification—metabolic pathway analysis—computational validation” strategy. First, UHPLC-Q-TOF-MS/MS technology is employed, integrated with online databases and reference standards, to systematically characterize its in vitro chemical composition. Second, by comparing mass spectrometry data from plasma and CSF samples, we screen and confirm the prototypical components and metabolites capable of entering the systemic circulation and penetrating the CNS. Finally, to bridge the gap between chemical identification and pharmacological efficacy, network pharmacology and molecular docking analyses are conducted to correlate the brain-penetrating metabolites with core pathological targets of ischemic stroke.

This study not only fills the research gap regarding the blood-entering components and in vivo transformation patterns of dragon’s blood resin, clarifying its core active substances responsible for treating cerebral ischemia, but also provides a reference technical paradigm for selecting quality control indicators for dragon’s blood resin (such as screening key blood-entering components) compound formulations for central nervous system disorders. It further establishes a technical paradigm for establishing “in vitro component-in vivo therapeutic substance” correlations. Additionally, it provides a scientific rationale for selecting quality control indicators for dragon’s blood resin (e.g., using key blood-penetrating components as quality markers) and for rational clinical application.

## 2. Materials and Methods

### 2.1. Reagents and Materials

The reagents used in this study included dragon’s blood (batch No. 190107, Xishuangbanna Jinglong Pharmaceutical Co., Ltd., Xishuangbanna, China) and Heparin (batch No. 210910, self-prepared with purity ≥ 98%). Reference standards of salicylic acid, resveratrol, trans-pterostilbene, loureirin A, esculetin, apigenin, oleanolic acid, biochanin A, and nobiletin (all purities ≥ 98% by HPLC) were purchased from Shanghai Taitan Technology Co., Ltd. (Shanghai, China). Carboxymethylcellulose Sodium (batch No. K1919043, Shanghai Aladdin Biochemical Technology Co., Ltd., Shanghai, China), Isoflurane (batch No. 21110601, Shenzhen RWD Life Science Co., Ltd., Shenzhen, China), Chromatographic Acetonitrile (batch No. X7MA1H, Honeywell, Charlotte, NC, USA), Chromatographic Formic Acid (batch No. D23251007, Shanghai Aladdin Biochemical Technology Co., Ltd., Shanghai, China), MCAO Line Embolus (251–330 g, batch No. P2475049, Shenzhen RWD Life Science Co., Ltd., Shenzhen, China), MCAO Line Embolus (251–280 g, batch No. P2475039, Shenzhen RWD Life Science Co., Ltd., Shenzhen, China), Medical Non-absorbable Suture (batch No. 20210805, Yangzhou Jinbao Medical Equipment Factory, Yangzhou, China), and T3 Chromatographic Column (batch No. 0294331671, Waters, Milford, MA, USA).

The instruments employed were an Analytical Balance (model DV215CD, OHAUS, Parsippany, NJ, USA), Small Animal Anesthesia Machine (model ABS-100, Shanghai Yuyan Scientific Instruments Co., Ltd., Shanghai, China), Low-temperature High-speed Centrifuge (model V18R, Dynamica, Livingston, UK), Vacuum Concentrator (model Bionoon-VAC1, Shanghai Bionoon Biotechnology Co., Ltd., Shanghai, China), Ultrahigh Performance Liquid Chromatography ((UHPLC, Nexera series, Shimadzu, Kyoto, Japan), and Quadrupole Time-of-Flight Mass Spectrometry Q/TOF-MS, model X500B QTOF, AB SCIEX, Framingham, MA, USA).

Male Sprague–Dawley (SD) rats weighing 220 ± 10 g were purchased from Beijing Spf Biotechnology Co., Ltd. (Beijing, China). All animal experiments were approved by the Animal Ethics Committee of the Yunnan Branch of the Institute of Medicinal Plant Development (IMPLAD), Chinese Academy of Medical Sciences (CAMS) and Peking Union Medical College (PUMC). All experimental procedures were conducted in strict accordance with the Group Standards and Implementation Guidelines issued by the Chinese Association for Laboratory Animal Sciences.

### 2.2. Preparation of Dragon’s Blood Extract

DB + B Group: Administered a combination of dragon’s blood (540 mg/kg) and Borneol (90 mg/kg). The 6:1 (*w*/*w*) dosing ratio of dragon’s blood to Borneol was chosen based on two primary considerations. First, it aligns with the clinical application of Borneol as a “guide drug” (Yin-Jing drug) in traditional Chinese medicine, where it is typically added in proportions of 10–20% relative to the primary medicine to facilitate targeted delivery. Second, this specific ratio was pharmacologically optimized in our previous study [9], which demonstrated its superior efficacy in reducing cerebral infarct volume and improving neurological scores in tMCAO rats compared to other combinations. The mixture was prepared using a geometric dilution method and suspended in 0.5% CMC-Na solution to a final volume of 10 mL/kg.

### 2.3. Animal Grouping and Drug Administration

The experimental procedures were conducted in healthy Sprague–Dawley rats to define the baseline brain-penetrating profile of dragon’s blood. This approach avoids the confounding effects of pathological BBB disruption characteristic of ischemic stroke, though it may underestimate the total CNS influx during a stroke event.

The rats were randomly divided into three groups: the Vehicle Control group (Blank, n = 5), the dragon’s blood group (DB, n = 5), and the Combination group (DB + B, n = 5, 4 valid CSF samples were obtained). Initially, five rats were used for CSF collection in each group (n = 5). However, one sample in the Combination group was excluded due to visible blood contamination during the puncture process, resulting in four valid samples (n = 4) for subsequent analysis to ensure data integrity. The dosing regimens were established as follows:

Vehicle Control Group: Administered 0.5% sodium carboxymethyl cellulose (CMC-Na) solution (10 mL/kg).

DB Group: Administered dragon’s blood suspension at a dose of 540 mg/kg (suspended in 0.5% CMC-Na, 10 mL/kg).

DB + B Group: Administered a combination of dragon’s blood (540 mg/kg) and Borneol (90 mg/kg). The mixture was prepared using a geometric dilution method to achieve a 6:1 (*w*/*w*) ratio of dragon’s blood to Borneol, which was then suspended in 0.5% CMC-Na solution to a final volume of 10 mL/kg.

All treatments were administered via oral gavage once daily for 7 consecutive days.

The relatively small sample size for CSF collection was determined based on the ethical guidelines for animal use reduction and the severe technical challenges associated with extracting sufficient, blood-free CSF from rats after transcardial perfusion. While constrained, this sample size provides necessary preliminary insights into CNS exposure.

### 2.4. Collection of Plasma and Cerebrospinal Fluid

Biological samples were collected 45 min after the final administration on the 7th day. Rats were anesthetized with isoflurane.

Plasma Collection:

Whole blood was collected from the abdominal aorta into heparinized tubes. The blood samples were immediately centrifuged at 3500 rpm for 15 min at 4 °C to separate the plasma. The supernatant (plasma) was transferred to clean tubes and stored at −80 °C until analysis.

Systemic Perfusion and CSF Collection:

To eliminate the interference of systemic blood on the metabolites detected in the central nervous system, rats were subjected to transcardial perfusion with 200 mL of ice-cold physiological saline via the left ventricle until the effluent from the right atrium was colorless. Subsequently, CSF was collected via cisterna magna puncture. A 30-gauge needle was carefully inserted into the depression between the occipital protuberance and the atlas vertebrae to draw the CSF. All CSF samples were immediately frozen in liquid nitrogen and stored at −80 °C.

Sample Preparation: 

The plasma and CSF samples were thawed slowly at 4 °C and vortex-mixed for 30 s. An aliquot of 80 μL of each sample (plasma or CSF) was transferred to a new centrifuge tube, and 320 μL of pre-cooled acetonitrile (containing internal standards) was added to achieve a 1:4 (*v*/*v*) ratio for protein precipitation. The mixture was vortexed thoroughly for 2 min and allowed to stand at 4 °C for 10 min. The samples were then centrifuged at 12,000 rpm for 15 min at 4 °C. The supernatant was carefully collected and evaporated to dryness using a vacuum centrifugal concentrator. The residue was reconstituted in 200 μL of an acetonitrile–water (1:1, *v*/*v*) mixture, vortexed for 1 min, and centrifuged again at 12,000 rpm for 10 min. The final supernatant was transferred to auto-sampler vials for UHPLC-Q-TOF-MS/MS analysis. A quality control (QC) sample was prepared by pooling 10 μL from each reconstituted sample to monitor the stability and reproducibility of the analytical system.

### 2.5. UHPLC-Q-TOF-MS/MS Analysis Conditions

Chromatographic separation was performed using Water ACQUITY UPLC BEH T3 column (2.1 mm × 100 mm, 1.8 μm) maintained at 35 °C. The mobile phases consisted of 0.1% formic acid in water (A) and 0.1% formic acid in acetonitrile (B). The flow rate was 0.3 mL/min, and the injection volume was 5 μL. The multistep linear gradient elution program was applied as follows: 0–2.0 min, 5% B; 2.0–3.0 min, 5–35% B; 3.0–8.0 min, 35–55% B; 8.0–13.0 min, 55–82.5% B; 13.0–14.0 min, 82.5–95% B; 14.0–17.0 min, 95% B (isocratic hold); 17.0–17.1 min, 95–5% B; followed by re-equilibration at 5% B from 17.1 to 21.0 min.

Mass spectrometry analysis was carried out using an ESI source in both positive and negative ion modes. Nitrogen was utilized as the curtain gas (CUR), nebulizer gas (GS1), heater gas (GS2), and collision gas (CAD). The source parameters were set as follows: ion source voltage, 4500 V (positive) and −4500 V (negative); source temperature, 550 °C; CUR, 35 psi; Gas 1, 60 psi; and Gas 2, 60 psi. The declustering potential (DP) was set at 80 V and −80 V for positive and negative modes, respectively. The collision energy (CE) was set at 40 V (positive) and −40 V (negative) with a spread of ±20 V. Data were acquired in Information Dependent Acquisition (IDA) mode. The scanning ranges were 60–1250 Da for MS1 and 50–1250 Da for MS2. The top 12 precursor ions with intensities greater than 100 cps were triggered for fragmentation.

### 2.6. Method Validation for Metabolomic

To ensure the reliability and reproducibility of the metabolomics analysis, method validation—including precision, repeatability, and stability—was evaluated using the pooled quality control (QC) samples. Instrumental precision was assessed by injecting the same QC sample six consecutive times. Method repeatability was evaluated by preparing and analyzing six parallel QC samples independently. Sample stability was tested by analyzing the same QC sample kept in the autosampler (4 °C) at 0, 8, 12, and 24 h. Furthermore, to ensure broad-spectrum metabolite extraction and minimize matrix effects, a standardized protein precipitation method using a 1:4 (*v*/*v*) ratio of sample to pre-cooled acetonitrile was optimized, and the robust clustering of QC samples in the PCA score plot was used to monitor the overall system stability.

### 2.7. Data Processing and Multivariate Statistical Analysis

The raw mass spectrometry data were processed using MS-DIAL software (version 4.90) for peak alignment, peak picking, deconvolution, and compound identification. To ensure the robustness of metabolite annotation, strict filtering parameters were applied: the MS1 mass tolerance was set at ±5 ppm, and the MS2 mass tolerance was set at ±10 ppm. Compound identification was performed by matching the precursor ion exact mass, MS/MS fragmentation patterns, and isotopic distribution ratios with public databases (e.g., MassBank, GNPS, and HMDB) integrated within MS-DIAL. A total identification score threshold of ≥80% (incorporating mass accuracy, isotopic similarity, and MS/MS dot product scores) was utilized to screen for reliable candidates. Only compounds meeting these criteria were considered putative identifications (MSI Level 2).

Subsequently, the generated peak intensity table, containing retention time, *m*/*z* pairs, and peak areas, was imported into the MetaboAnalyst 6.0 platform (https://www.metaboanalyst.ca/, accessed on 7 May 2026) for multivariate statistical analysis [11], including Principal Component Analysis (PCA) and Orthogonal Partial Least Squares Discriminant Analysis (OPLS-DA), to evaluate the distribution efficacy and metabolic shifts across different biological matrices.

### 2.8. Molecular Docking Protocol

Molecular docking was performed using AutoDock Vina (version 1.2.0). The 3D structures of the proteins (TNF-α: 2AZ5; PTGS2: 5IKQ; AKT1: 4EJN) were obtained from the PDB database and prepared by removing water molecules and adding polar hydrogens using AutoDockTools (version 1.5.6). Protonation states were assigned at pH 7.4. The grid box was centered on the original ligand binding site with dimensions of 25 Å × 25 Å × 25 Å and a spacing of 0.375 Å. The exhaustiveness parameter was set to 32 to ensure thorough sampling. The best-scoring pose (lowest Vina score in kcal/mol) was selected for analysis.

## 3. Results

### 3.1. Chemical Analysis of Dragon’s Blood Extract (UHPLC-Q-TOF-MS/MS Analysis)

**Figure 1:**
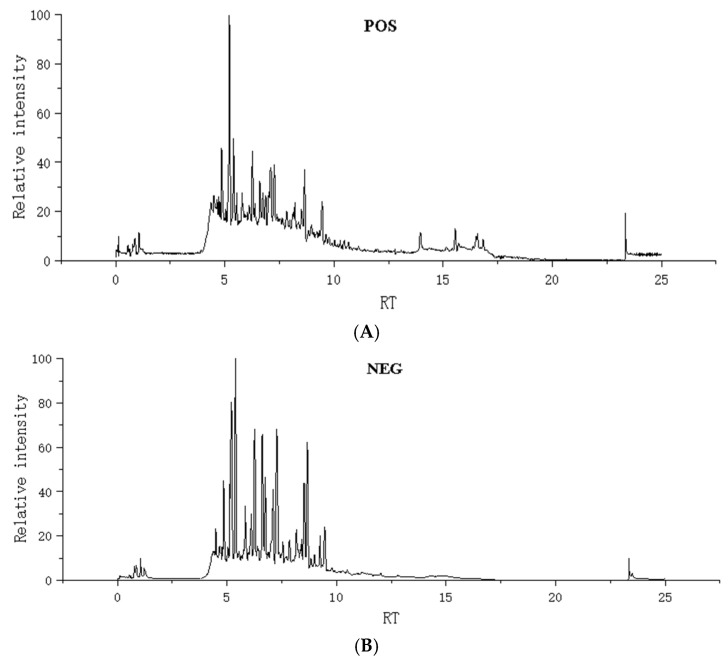
Representative UHPLC-Q-TOF-MS/MS total ion chromatograms (TIC) of dragon’s blood extract. (**A**) Positive ion mode (ESI+); (**B**) negative ion mode (ESI−). The main peaks correspond to the characteristic flavonoids and steroids characterized in Table S1.

The chemical constituents of dragon’s blood resin extract were analyzed using UHPLC-Q-TOF-MS/MS. Under optimized UHPLC-Q-TOF-MS/MS conditions, the total ion current (TIC) chromatograms of the extract in negative and positive ion modes are shown in Figure 1.

**Figure 2:**
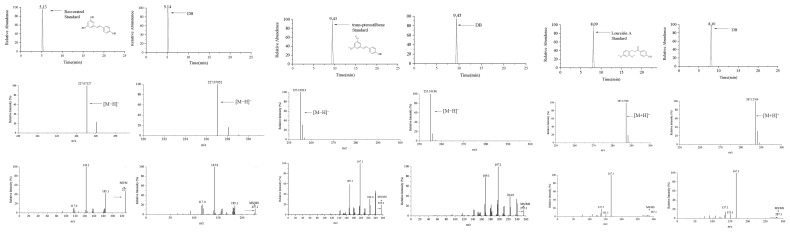
Compound identification based on comparison with reference standards. (*) denote precursor ions in the MS/MS spectra.

In this study, nine reference substances (salicylic acid, resveratrol, trans-pterostilbene, loureirin A, esculetin, apigenin, oleanolic acid, biochanin A, and nobiletin) were employed to ensure the accuracy of the identification. Figure 2 illustrates the MS/MS spectral comparison of three representative components: resveratrol, trans-pterostilbene, and loureirin A. Other prototype components confirmed by their respective reference standards are specifically marked with an asterisk (*) in Supplementary Table S1.

For example, the structural elucidation of loureirin A (peak 64; molecular formula: C_17_H_18_O_4_) was achieved in the positive ion mode. As shown in Figure 2, the extracted ion chromatogram displayed a distinct peak at a retention time of 8.33 min. The precursor ion was observed at *m*/*z* 287.1278 [M+H]^+^, which is highly consistent with the theoretical accurate mass of protonated loureirin A. In the MS^2^ spectrum, the parent ion underwent characteristic fragmentation, yielding abundant product ions. The most dominant fragment was observed at *m*/*z* 167.0700 (base peak), along with other typical diagnostic ions at *m*/*z* 151.0754, 137.0593, and 124.0500. The retention time, accurate mass of the precursor ion, and the unique MS^2^ fragmentation pattern of peak 64 were strictly identical to those of the authentic reference standard, thereby unambiguously confirming its identity as loureirin A (MSI Level 1).

**Figure 3:**
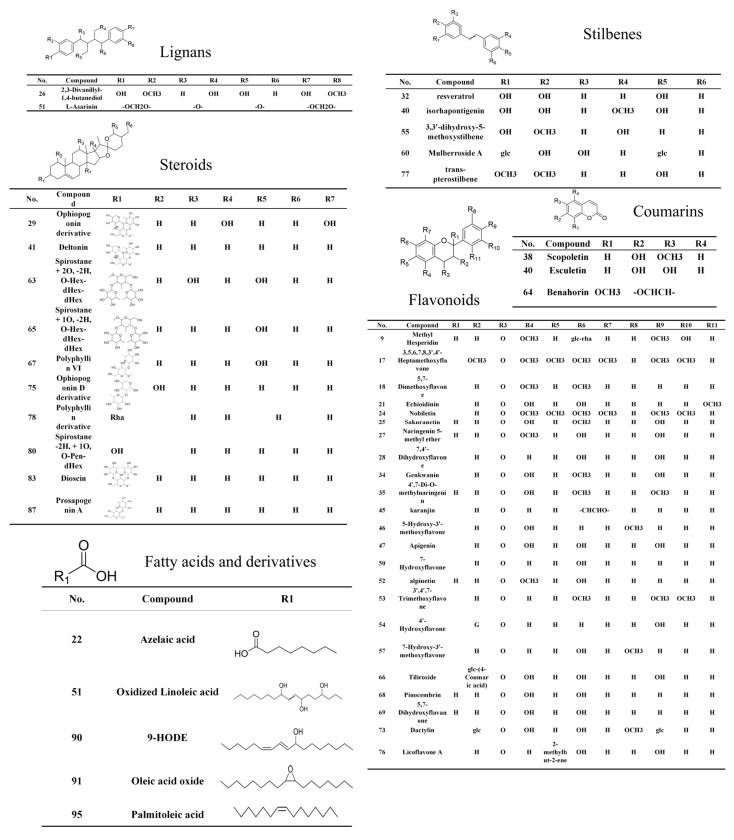
Classification of the 96 constituents identified in dragon’s blood resin.

**Figure 4:**
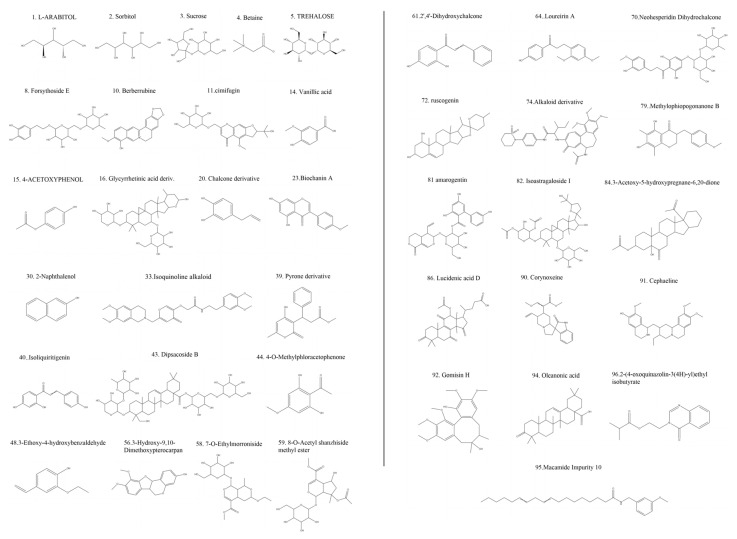
Chemical structures of all 96 constituents characterized in dragon’s blood.

Based on the retention time, accurate mass ion, and comparison of MS^2^ fragments with reference substances, 9 compounds in dragon’s blood resin were accurately identified. Furthermore, a total of 96 compounds were characterized based on HRMS data and database matching, including 31 flavonoids, 14 steroids, 12 terpenes, 5 fatty acids, 4 styrenes, 4 coumarins, 3 isoflavones, and 25 other constituents (Figure 3 and Figure 4).

#### 3.1.1. Flavonoids

**Figure 5:**
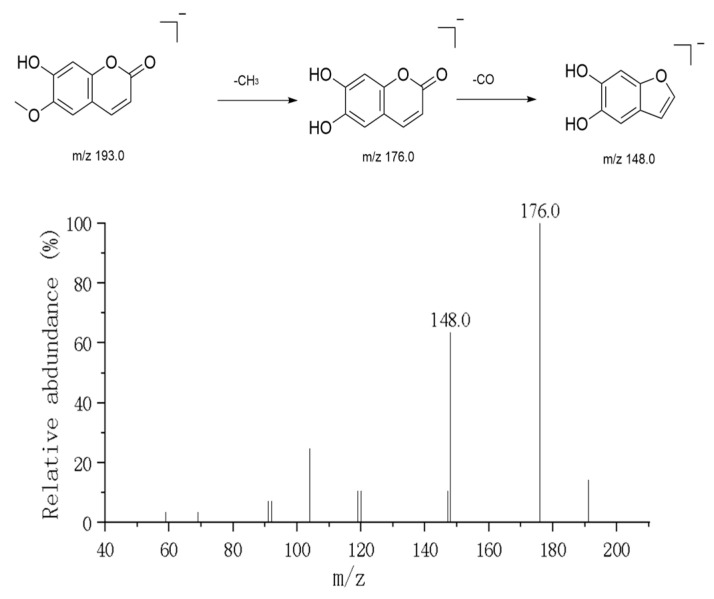
Mass spectrometric analysis of apigenin.

Flavonoids are the primary active constituents of dragon’s blood resin. Under negative ion mode, these compounds readily undergo Retro-Diels–Alder (RDA) cleavage. The structure of flavonoids consists of a basic skeleton (C6-C3-C6), where two benzene rings (ring A and ring B) are connected via a three-carbon heterocyclic ring (ring C). Their fragmentation patterns primarily depend on whether they are aglycones or glycosides, as well as the type and position of substituents (e.g., hydroxyl, methoxy groups). Typically, mass spectrometry fragmentation of flavonoid aglycones primarily involves Retro-Diels–Alder (RDA) reactions [12], along with neutral loss of small molecules like H_2_O (18 Da) and CO (28 Da). For flavonoids bearing methoxy substituents, the loss of a methyl radical (CH_3_•, 15 Da) represents a characteristic fragmentation pathway. Based on these fragmentation patterns and reference standard comparisons, a total of 34 flavonoid components were identified. For example, the [M-H]^−^ ion of Apigenin [13] exhibits a strong signal at *m*/*z* 269.0446 for the [M-H]^−^ ion, with a predicted molecular formula of C_15_H_9_O_5_^−^. In the MS^2^ spectrum, its fragmentation pattern follows typical flavonol aglycone behavior. The parent ion undergoes classic RDA fragmentation, yielding key fragment ions corresponding to rings A and B. Specifically, the fragment ion at *m*/*z* 151.0036 is assigned as the [1,3A]^−^ product containing ring A, while the fragment ion at *m*/*z* 117.0345 corresponds to the [1,3B]^−^ product containing ring B. Additionally, an ion at *m*/*z* 241.0502 was observed, formed by the loss of a CO molecule from the parent ion. These fragmentations collectively confirm the structure of 5,7,4′-trihydroxyflavone (Figure 5).

**Figure 6:**
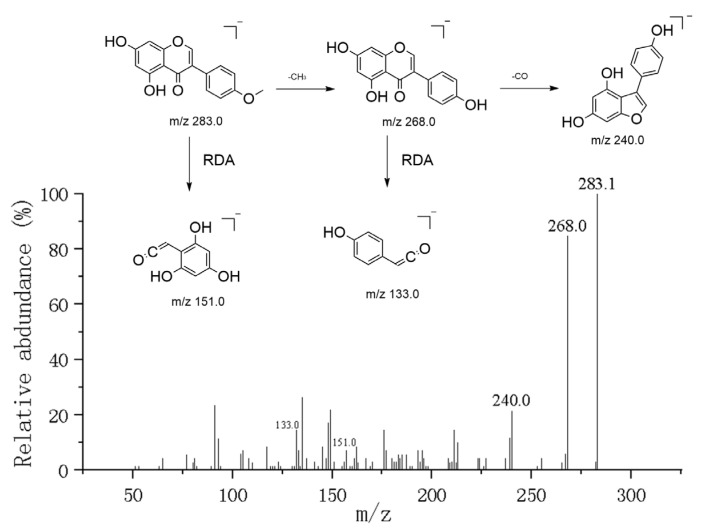
Mass spectrometric analysis of Biochanin A.

Unlike apigenin (a flavone), biochanin A belongs to the isoflavone class, with its precursor ion peak at *m*/*z* 283.0609 ([M-H]^−^). Influenced by the 4′-methoxy group on the B-ring, this compound exhibits unique fragmentation characteristics: its dominant fragmentation pathway involves radical homolytic cleavage, preferentially removing a methyl radical (•CH_3_, 15 Da) to generate a highly abundant radical anion at *m*/*z* 268.0378 ([M-H-•CH_3_]•^−^). This demethylated intermediate subsequently undergoes further secondary fragmentation. Unlike the fragmentation patterns of typical flavonoids, the characteristic fragment *m*/*z* 132.0227 observed in Biochanin A is attributed to a Retro-Diels–Alder (RDA) fragmentation product from the demethylated B-ring portion (^1′3^B-ring-related fragment). Additionally, owing to its A-ring bearing the same 5,7-dihydroxy substitution pattern as apigenin, the characteristic fragment *m*/*z* 151.0006, assigned to ^1′3^A^−^, was also observed in the spectrum (Figure 6).

The compound Tiliroside eluting at 15.8 min exhibits an [M-H]^−^ ion at *m*/*z* 595.1359. Its characteristic fragmentation involves the loss of an intact p-coumaroyl-glucoside residue (310 Da), directly yielding the product ion at *m*/*z* 285.0408. The observed *m*/*z* 429.1162 ion corresponds to a product where the parent ion loses only a glucose moiety (162 Da), indicating that both the p-coumaroyl and glucose groups are jointly attached to the aglycone. The *m*/*z* 285.0408 base ion and its subsequent RDA fragmentation patterns are characteristic mass spectrometry features of kaempferol. Based on the above information, the compound was preliminarily identified as tiliroside [14], a previously reported kaempferol-3-O-(6″-O-p-coumaroyl)-β-D-glucopyranoside.

#### 3.1.2. Steroid

Overall, 8 of the 12 steroid compounds are steroidal saponins. The mass spectrometry fragmentation of steroidal saponins typically follows a sequential loss pattern of their sugar chains until aglycone ions are formed, whereas the fragmentation of steroid aglycones primarily involves characteristic cleavages of the steroid ring skeleton. The compound Deltonin [15] detected in chromatographic analysis exhibits a precursor ion peak at *m*/*z* 907.4679 (predicted molecular formula C_45_H_71_O_17_^−^). In secondary mass spectrometry, its most characteristic cleavage involves the loss of the glucose residue at the C-26 position (162 Da), yielding a product ion at *m*/*z* 745.2709. This signature neutral loss not only confirms its furostanol saponin structure but also indicates that this fragment corresponds to the spirostanol saponin structure formed by the cyclization of its side chain.

#### 3.1.3. Terpenoids

Additionally, several terpenoids were identified or characterized in this study. Among them, Oleanonic acid and ruscogenin, two common triterpenoids, were preliminarily identified through database comparison [16,17]. Oleanonic acid exhibited a precursor ion peak at *m*/*z* 455.35 [M+H]^+^ in mass spectrometry, while ruscogenin displayed a sodium adduct peak at *m*/*z* 453.30 [M+Na]^+^. Their secondary mass spectra exhibited similar fragmentation patterns, primarily involving sequential neutral loss of H_2_O molecules.

Additionally, the compound Agnuside was characterized as an iridoid glycoside through spectral matching with the MassBank database. Its mass spectrometry analysis revealed an [M+Na]^+^ peak at *m*/*z* 489.13, with a characteristic fragmentation pattern involving the loss of the sugar moiety (162 Da), typical of glycoside compounds. Similarly, Dipsacoside B was identified as a complex triterpene saponin based on its characteristic mass spectrometric behavior. Its MS/MS fragmentation pattern revealed the sequential cleavage of multiple glycosidic bonds, resulting in the stepwise shedding of sugar chains.

#### 3.1.4. Coumarin

**Figure 7:**
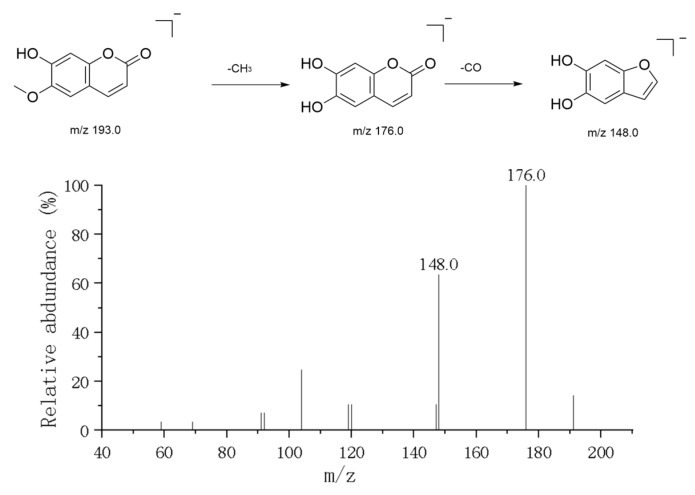
Mass spectrometric analysis of Scopoletin.

In positive ion mode, two major simple coumarin components were detected: Esculetin and Scopoletin. The precursor ion peak for Esculetin was observed at *m*/*z* 179.03 ([M+H]^+^). Its secondary mass spectrum (MS^2^) exhibited the characteristic fragmentation pattern of 2-dihydroxycoumarin: First, a water molecule is lost to produce the characteristic fragment ion at *m*/*z* 161.02, indicating the presence of an unstable hydroxyl group in its structure. Subsequently, or directly, the αα-pyranone ring undergoes cleavage, losing a CO_2_ molecule to form the fragment ion at *m*/*z* 135.01, which is the most distinctive mass spectrometry behavior characteristic of the coumarin skeleton [18]. Additionally, further decarboxylation (COCO) fragments such as *m*/*z* 133 and *m*/*z* 105 are observed. Scopoletin exhibits a parent ion peak at *m*/*z* 193.05 ([M+H]^+^). Compared to Esculetin, its cleavage pathway differs slightly due to the presence of a methoxy substitution at the C-6 position in its structure (Figure 7). Fragments such as *m*/*z* 176 and *m*/*z* 148 were observed in the MS2 spectrum, typically involving the loss of a methyl radical or methoxy group followed by carbonyl elimination. Additionally, a strong signal at *m*/*z* 307.09 was detected within the same chromatographic retention time window. Its secondary mass spectrum showed only retention of the parent ion (*m*/*z* 307.07) with minimal fragment production, suggesting this ion represents an extremely stable sodium adduct [M+Na]^+^. The neutral molecular weight is inferred to be 284 Da. This component may be a phenolic compound with a molecular weight of 284 coexisting with coumarin (e.g., acacetin), which readily forms sodium adducts in the electrospray ionization source. The coordination of sodium ions enhances molecular stability, making it difficult for the backbone to undergo fragmentation under conventional collision energies.

#### 3.1.5. Fatty Acids and Their Derivatives

In this study, a series of fatty acids and their derivatives were identified based on their secondary mass spectrometry fragmentation patterns. These compounds typically exhibit neutral losses such as water (H_2_O) and carbon dioxide (CO_2_) in mass spectrometry analysis. For example, a linoleic acid derivative was tentatively identified, exhibiting a precursor ion peak [M-H]^−^ at *m*/*z* 295.2280 in the mass spectrum. Its primary fragment ion peak was observed at *m*/*z* 277.2169, formed by the removal of one water molecule (18 Da) from the parent ion, a cleavage pattern characteristic of hydroxylated fatty acids [19].

Additionally, other fatty acid compounds were identified based on their MS2 fragment spectra, including long-chain fatty acids and oxidized fatty acids. Among these, one compound was tentatively identified as an epoxy fatty acid, exhibiting a parent ion peak at [M-H]^−^ *m*/*z* 297.2432.

#### 3.1.6. Diphenylstyrene Compounds

**Figure 8:**
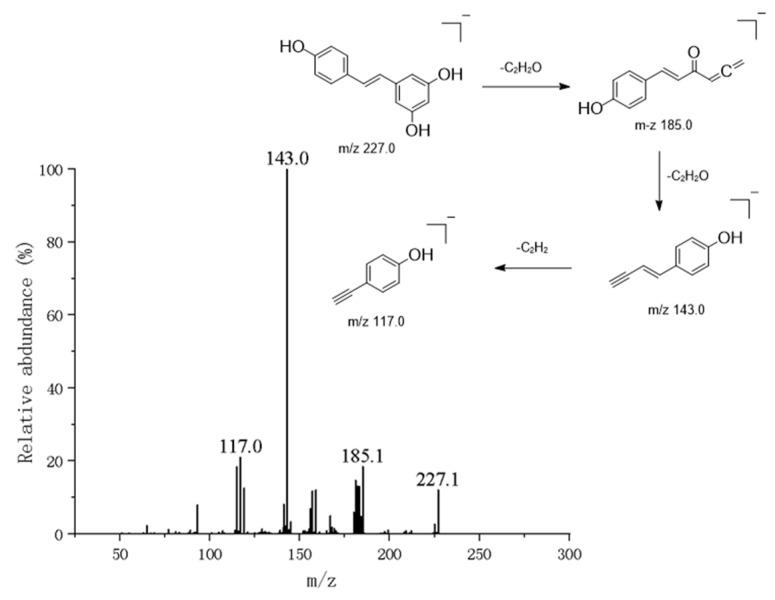
Mass spectrometric analysis of resveratrol.

Additionally, some diphenylstyrene compounds identified or characterized in this study have a molecular weight of 180 and a structure of C_6_H_5_-CH=CH-C_6_H_5_. Due to their highly stable conjugated systems, their molecular ion peaks are typically strong, often appearing as base peaks. When substituents are present on the benzene rings, the fragmentation patterns change. The nature and position of substituents dictate new fragmentation pathways [20]. For example, resveratrol, a core diphenyl ethylene compound, was identified (Figure 8). In negative ion mode, it yields a precursor ion peak at *m*/*z* 227.0706 [M-H]^−^ [21]. Its secondary mass spectrum exhibits characteristic fragmentation patterns, with primary fragment ions at *m*/*z* 185.0601 and *m*/*z* 143.0495, primarily attributed to cleavage of the central ethylene bridge. Structural confirmation was achieved through a literature comparison.

Concurrently, isorhapontigenin—a methoxylated derivative of resveratrol—was identified. Its [M-H]^−^ peak at *m*/*z* 257.0814 exhibited characteristic mass-spectral fragmentation at *m*/*z* 185.0601, indicating cleavage of the resorcinol ring (Ring A). Its characteristic *m*/*z* 185.0601 mass spectrometry fragmentation peak represents an intermediate formed by ring-opening cleavage of the resorcinol ring (Ring A), resulting in the loss of one ethylenone molecule. This intermediate subsequently undergoes further fragmentation of the phenolic ring at *m*/*z* 257.0814.

### 3.2. Multivariate Statistical Analysis of CSF Metabolome

**Figure 9:**
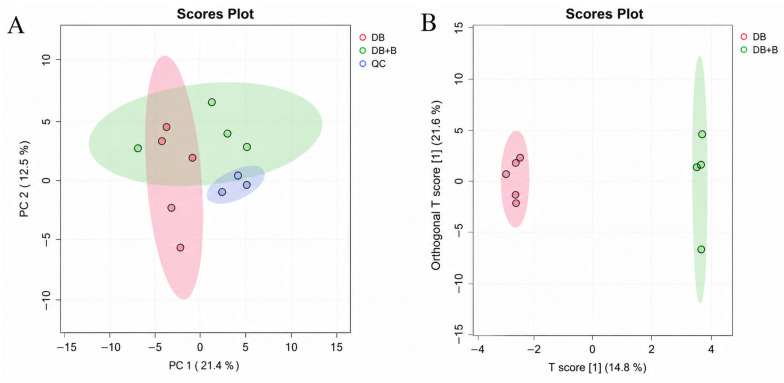
Multivariate statistical analysis of CSF metabolomic profiles (ESI+ mode). (**A**) PCA score plot of all samples, including QC samples (Blue), dragon’s blood group (DB, Red), and Combination group (DB + B, Green). (**B**) OPLS-DA score plot showing clear separation between the dragon’s blood group and Combination group.

To systematically evaluate the metabolic alterations induced by Borneol, an untargeted metabolomic approach was applied to the CSF samples. The stability of the UPLC-Q-TOF-MS system was first assessed using Principal Component Analysis (PCA). As shown in Figure 9A, the quality control (QC) samples (blue dots) were tightly clustered in the center of the score plot, indicating excellent reproducibility and instrumental stability throughout the analytical run. This confirms that the observed metabolic variations were primarily biologically derived rather than caused by technical errors.

Subsequently, a supervised Orthogonal Partial Least Squares Discriminant Analysis (OPLS-DA) was performed to maximize the discrimination between the dragon’s blood group (n = 5) and the Combination group (n = 4). As illustrated in Figure 9B, the two groups exhibited a clear and robust separation along the first principal component (*x*-axis, T score), suggesting that the co-administration of Borneol induced distinct global metabolic shifts in the CSF. The model parameters indicated high explanatory power (R2Y = 0.743) and acceptable predictive capability (Q2 = 0.604), supporting the statistical significance of the metabolic differences. Furthermore, a 200-iteration permutation test was conducted to validate the robustness of the OPLS-DA model and rule out overfitting. The resulting intercepts (R2 = (0.0,0.321), Q2 = (0.0,−0.062)) confirmed that the original model was statistically reliable and not overfitted (Supplementary Figure S1).

The method validation results demonstrated the high reliability of the analytical system. For representative high-, medium-, and low-abundance ions in the QC samples, the relative standard deviations (RSDs) of retention times and peak areas for instrumental precision were less than 0.5% and 6.5%, respectively. The RSDs for method repeatability were all below 8.5%. Furthermore, the samples exhibited excellent stability in the autosampler over 24 h, with peak area RSDs remaining below 9.0%. These validation parameters, coupled with the tight clustering of QC samples in the center of the PCA score plot (Figure 9A), confirm that the variations observed in the biological samples are entirely driven by the physiological and pharmacological interventions rather than analytical or matrix artifacts.

### 3.3. Comparative Analysis of Blood and Cerebrospinal Fluid Metabolomes

To evaluate the central nervous system (CNS) exposure of dragon’s blood constituents and the synergistic effect of Borneol, a comparative metabolomic analysis of plasma and cerebrospinal fluid (CSF) was conducted. This approach differentiates between compounds that are directly absorbed, peripherally metabolized, and those capable of penetrating the blood–brain barrier (BBB). A summary of representative prototypes and their metabolic fates is provided in Table 2.

#### 3.3.1. Systemic Exposure and Peripheral Biotransformation (Blood Analysis)

Analysis of the plasma samples (DB group) revealed that several prototypes were directly absorbed into the systemic circulation without transformation, including amino acid derivatives (Betaine), lignans (Gomisin H), anisoles (Macamide Imp 10), and fatty acids (Palmitoleic acid).

Simultaneously, extensive peripheral biotransformation was observed. The primary metabolic pathways in plasma involved fatty acid chain elongation (e.g., Palmitoleic acid to Oleic acid) and Phase I modifications such as O-demethylation (e.g., Nobiletin to Artemitin) and hydroxylation (e.g., Oleic acid epoxide to its 8-hydroxy-derivative).

Notably, co-administration with Borneol significantly enriched the systemic metabolic profile (DB + B group). Extensive deglycosylation of isoflavones and stilbenes occurred in the blood; for instance, Dactylin and Mulberroside A were hydrolyzed into their respective aglycones (Daidzein, Daidzin). Furthermore, Biochanin A underwent Phase I demethylation to yield Genistein. This indicates that Borneol enhances the formation of lipophilic aglycones in the systemic circulation.

#### 3.3.2. CNS Distribution and BBB-Penetrating Metabolites (CSF Analysis)

The CSF metabolome represents the fraction of circulating compounds that permeate the BBB. Our data demonstrated that highly polar, large-molecular-weight parent glycosides were largely absent in the CSF, validating a “pro-drug” mechanism where peripheral hydrolysis is a prerequisite for brain penetration.

Prototypes that directly penetrated the BBB included Betaine, Gomisin H, and Macamide Imp 10. A striking observation in the CSF was the convergent metabolism of stilbenoids. Multiple parent stilbenes—including Isorhapontigenin, Mulberroside A, Resveratrol, and 3,3′-dihydroxy-5-methoxyresveratrol—were all ultimately detected in the CSF as Oxyresveratrol. This funneling effect involves sequential deglycosylation, demethylation, and hydroxylation. Given Oxyresveratrol’s potent antioxidant properties, this finding provides a strong material basis for the efficacy of dragon’s blood in ischemic stroke.

#### 3.3.3. Putative Deep Metabolic Modulation Mediated by Borneol Synergy

Metabolomic mapping suggested that Borneol potentially modulates the in vivo fate of dragon’s blood constituents beyond physical permeation enhancement. In the CSF of the combination group, specific “non-intuitive” transformations were putatively observed. For example, 4-Hydroxybenzaldehyde is proposed to undergo dehydroxylation to yield Benzaldehyde. Furthermore, we observed complex skeletal rearrangements, such as the cyclization of Berberrubine to the alkaloid Coptisine, and the transformation of Dactylin to Cyanidin-3-glucoside chloride.

These findings lead to the hypothesis that Borneol potentially alters metabolic enzyme activities (e.g., CYP3A/1A/2D subfamilies) or gut microbiota interactions, shifting the metabolic flux toward highly bioactive small molecules in the CNS. However, as these mechanisms were not directly assessed in this study, they represent important directions for future experimental validation.

#### 3.3.4. Semi-Quantitative Analysis of Borneol-Driven Enrichment

**Figure 10:**
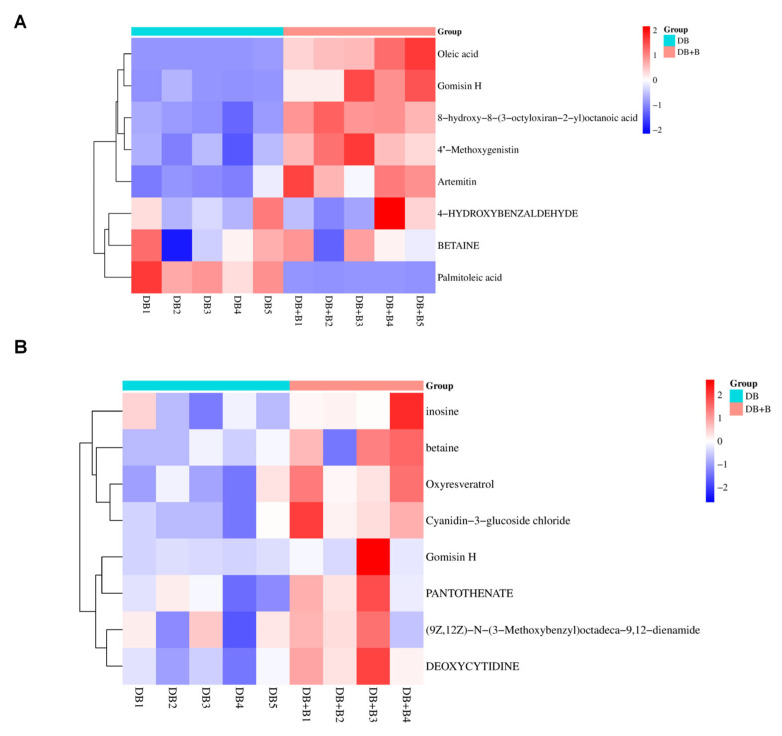
Hierarchical clustering and heatmap visualization of metabolite distribution efficacy. (**A**) Heatmap of plasma metabolites; (**B**) heatmap of CSF metabolites.

Hierarchical clustering and heatmap visualization were performed to evaluate the distribution efficacy (Figure 10).

In the systemic circulation (Figure 10A), the heatmap showed that Borneol significantly upregulated the relative abundance of parent compounds and Phase I metabolites like Gomisin H, Artemitin, and 4″-Methoxygenistin. A shift in lipid profiles was also captured: Palmitoleic acid was abundant in the DB group, while its downstream product, Oleic acid, was heavily enriched in the DB + B group, confirming that Borneol accelerates systemic lipid metabolism.

In the central nervous system (Figure 10B), the CSF heatmap confirmed Borneol’s capacity to enhance CNS exposure. Exogenous metabolites—most notably Oxyresveratrol, Coptisine, and Gomisin H—displayed significantly higher relative abundances in the combination group. Furthermore, the combination therapy upregulated essential endogenous neuromodulators in the CSF, such as Inosine and Pantothenate, indicating that Borneol facilitates the entry of drug effectors while synergistically restoring the metabolic microenvironment of the ischemic brain.

### 3.4. Network Pharmacology Analysis of Brain-Penetrating Metabolites

To further elucidate the therapeutic mechanism of the identified metabolites in treating ischemic stroke, a systematic network pharmacology approach was employed based on the eight core exogenous metabolites (e.g., Oxyresveratrol, Gomisin H, Coptisine, and Artemitin) detected in the CSF.

#### 3.4.1. Identification of Overlapping Targets and PPI Network

**Figure 11:**
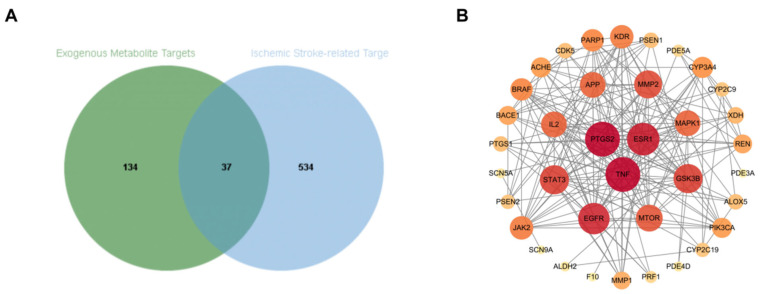
Target overlap analysis and protein–protein interaction network of brain-penetrating metabolites against ischemic stroke. (**A**) Venn diagram showing the overlapping targets between 8 core CSF metabolites and ischemic stroke; (**B**) protein–protein interaction (PPI) network of the 34 common hub targets.

A total of 159 candidate targets were predicted for the eight CSF metabolites, which were then intersected with 571 high-confidence targets associated with ischemic stroke. As shown in Figure 11A (Venn diagram), 34 common targets were identified.

Topological analysis of the protein–protein interaction (PPI) network was performed to pinpoint the hub genes (Figure 11B). Among the 34 common targets, PTGS2 (COX-2) and TNF exhibited the highest node degrees (degree = 24), followed by AKT1 (degree = 19). These results suggest that the neuroprotective effect of dragon’s blood and Borneol is primarily mediated through the modulation of these central hubs involved in neuroinflammation and cell survival.

#### 3.4.2. KEGG Pathway Enrichment Analysis

**Figure 12:**
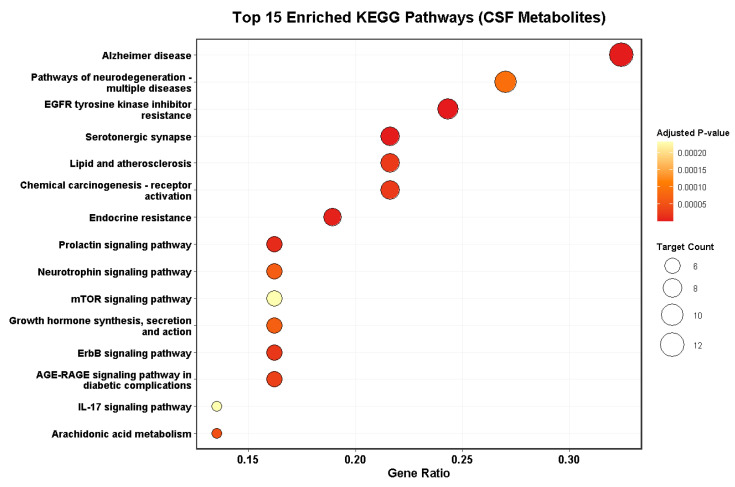
KEGG pathway enrichment analysis of common targets.

To explore the signaling pathways regulated by these 34 targets, KEGG enrichment analysis was performed (Figure 12). The core targets were significantly enriched in several pathways critical to stroke pathology:

Arachidonic acid metabolism: This pathway, involving the hub gene PTGS2, is central to post-ischemic inflammation and brain edema.

Neurotrophin and mTOR signaling pathways: These pathways are crucial for neuronal survival, autophagy regulation, and neurorepair in the ischemic penumbra.

Pathways of neurodegeneration: The enrichment in Alzheimer’s and general neurodegeneration pathways (involving targets like APP, BACE1, and PSEN1) underscores the potential of these metabolites to mitigate long-term cognitive decline following stroke.

### 3.5. Molecular Docking Validation of Core Interactions

To provide structural evidence for the predicted mechanisms, molecular docking was performed between the star CSF metabolites and the primary hub targets identified in the PPI network.

**Figure 13:**
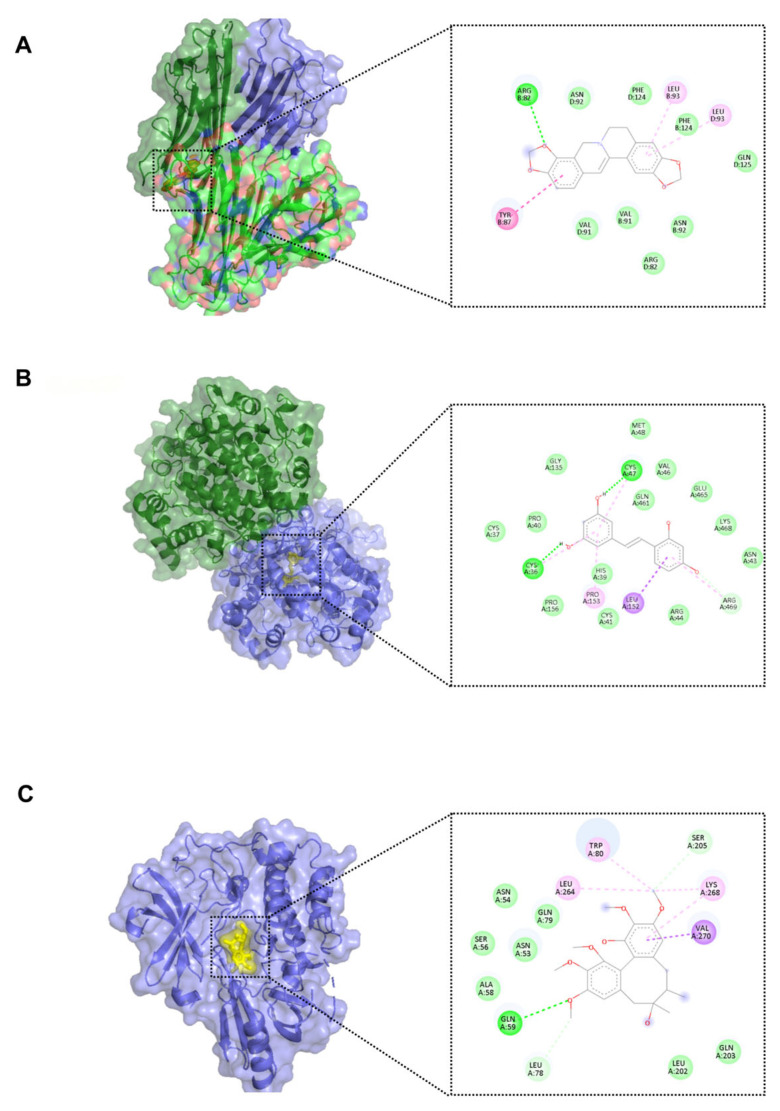
Molecular docking validation of core interactions. (**A**) Coptisine-TNF-α complex; (**B**) Oxyresveratrol-PTGS2 (COX-2) complex; (**C**) Gomisin H-AKT1 complex.

As summarized in Table 3, all tested pairs exhibited robust binding affinities. Notably, Coptisine demonstrated a favorable docking pose and high affinity for TNF-α (Vina score: −9.2 kcal/mol), stabilized by key hydrogen bonding with ARG-82 (Figure 13A). Similarly, Oxyresveratrol, the central product of convergent metabolism in the CNS, formed a stable complex with PTGS2 (Vina score: −8.3 kcal/mol) through anchoring hydrogen bonds with CYS-36 and CYS-47 (Figure 13B). Furthermore, Gomisin H exhibited significant affinity for AKT1 (Vina score: −6.6 kcal/mol), supporting its role in survival signaling (Figure 13C). These in silico findings support the material basis of dragon’s blood, suggesting a potential for these constituents to bind and modulate the core regulatory proteins.

To contextualize these docking results, the potent small-molecule TNF-α inhibitor SPD304 was docked as a positive control under identical parameters (detailed docking data and visualizations are provided in Supplementary Materials, Figure S2). SPD304 yielded a Vina score of −9.6 kcal/mol, while Coptisine exhibited a highly competitive score of −9.2 kcal/mol. This comparable binding affinity to a specialized inhibitor suggests that Coptisine possesses a strong potential for stable interaction within the target’s active pocket, potentially contributing to its neuroprotective effects.

## 4. Discussion

### 4.1. Characterization of the Material Basis of Dragon’s Blood for CNS Delivery

The therapeutic efficacy of traditional Chinese medicine (TCM) in treating central nervous system (CNS) diseases is often limited by the blood–brain barrier (BBB). In this study, we successfully established a comprehensive chemical library of dragon’s blood using UHPLC-Q-TOF-MS/MS. The identification of 96 compounds, including flavonoids, stilbenes, and saponins, provides a solid material basis for its multi-target pharmacological effects. Consistent with previous studies [22], flavonoids were identified as the dominant components. However, our study goes further by strictly screening for components capable of penetrating the BBB. It is important to note that while the presence of these compounds in the CSF is a widely accepted proxy for CNS exposure, it does not strictly differentiate between compounds crossing the endothelial blood–brain barrier (BBB) versus the blood–CSF barrier (BCSFB) at the choroid plexus. Nevertheless, their detection in the CSF strongly supports their availability to act on central neural targets.

By comparing the CSF profiles of the dosing group versus the control group, we confirmed that only specific lipophilic small molecules and metabolites could reach the brain, challenging the assumption that all absorbed components are bioactive in the CNS [23]. Interestingly, although steroids and terpenoids were abundant in the in vitro extract, they were largely absent in the CSF. This exclusion may be attributed to their relatively large molecular size and extreme lipophilicity, which favors their partitioning into circulatory lipoproteins rather than the aqueous CSF. Furthermore, many steroidal saponins are known substrates for P-glycoprotein (P-gp) efflux pumps at the BBB, which may actively limit their central accumulation. Our findings underscore the necessity of analyzing CSF markers, rather than relying solely on plasma pharmacokinetics, when elucidating the neuroprotective material basis of TCMs.

The brain-penetrating profile identified in this study is consistent with findings from similar metabolomics-based investigations into traditional Chinese medicines, such as Ginkgo biloba and Salvia miltiorrhiza, where specific lipophilic constituents were screened as key CNS effectors. Notably, the detection of resveratrol and trans-pterostilbene in the CSF serves as a benchmark for our results, as these compounds possess well-documented BBB permeability in previous pharmacokinetic studies [24,25]. By aligning our untargeted metabolomics findings with these established BBB-penetrant standards, we provide additional validation for the reliability of using CSF markers to decipher the material basis of dragon’s blood. However, it should be noted that under ischemic conditions, the BBB’s structural integrity is compromised, typically leading to increased permeability. Therefore, the metabolites identified in our healthy rat model likely represent the ‘minimum’ material basis, which would be further enriched in the pathological state of a stroke patient.

### 4.2. Metabolic Bioactivation: The “Pro-Drug” Mechanism and Antioxidant Enhancement

A central finding of this research is the proposal of a multi-step metabolic transformation network—specifically involving demethylation, glycoside hydrolysis, and oxidation—that facilitates the central delivery of dragon’s blood constituents [26].

Firstly, we observed a strikingly convergent metabolism among stilbenoids, where multiple parent structures (e.g., isorhapontigenin, mulberroside A, and resveratrol) were putatively funneled into oxyresveratrol within the CNS. While methylation typically enhances lipophilicity and intestinal absorption, subsequent demethylation in the liver or brain restores free hydroxyl groups. Given that these hydroxyl groups are essential for scavenging reactive oxygen species (ROS), this conversion suggests a “carrier–effector” bioactivation mechanism: parent compounds act as systemic carriers to bypass initial barriers, while their de-methylated metabolites function as the potent effectors against ischemic oxidative stress. This hypothesis is supported by existing pharmacological evidence, where oxyresveratrol has been independently validated to exert neuroprotection by inhibiting the NF-κB and MAPK pathways in ischemic models [27], providing functional plausibility to its role as a central effector.

Secondly, our results provide modern chemical support for the “pro-drug” hypothesis of saponins and glycosides [28]. The predominant detection of aglycones (e.g., diosgenin) alongside the near-total absence of their parent glycosides in the CSF indicates that peripheral hydrolysis—mediated by intestinal microbiota or endogenous glycosidases—is a prerequisite for BBB penetration. Although polar sugar moieties hinder BBB transport, they may enhance the initial solubility and bioavailability of the resinous components in the gastrointestinal tract before being cleaved. By aligning our metabolic findings with the well-documented neuroprotective activities of these aglycones, this study clarifies the chemical identity of the substances actually responsible for the clinical efficacy of dragon’s blood in CNS therapy.

### 4.3. The Synergistic Mechanism of Borneol: Beyond Physical Permeation

Borneol is classically regarded as a “guide drug” (or Yin-Jing drug) in TCM [29], believed to enhance the delivery of other therapeutics to the upper body and brain. Our metabolomic data provides modern chemical evidence for this theory, revealing two distinct mechanisms:

Enhanced Distribution: We confirmed via semi-quantitative heatmap analysis that Borneol co-administration significantly increases the relative abundance of lipophilic flavonoids (e.g., Gomisin H, Artemitin) in both plasma and CSF. This is likely achieved by transiently opening the tight junctions of the BBB [30], a physical permeation enhancement consistent with the literature.

More importantly, our study uncovered a previously underappreciated chemical interaction. Borneol not only increased the influx of prototypes but profoundly altered their in vivo fate. In the combination group, specific “non-intuitive” transformations emerged, such as the cyclization of Berberrubine to the highly bioactive alkaloid Coptisine. This suggests that Borneol may modulate the activity of hepatic or cerebral metabolic enzymes (such as CYP450s) [31] or alter gut microbiota dynamics, shifting the metabolic flux toward small molecules that are highly compatible with cerebral transport systems.

Specifically, the metabolic cyclization of Berberrubine to Coptisine observed in the combination group leads to the hypothesis that Borneol may modulate the activity of specific hepatic/cerebral CYP450 enzymes or shift the gut microbiota equilibrium. It must be explicitly stated, however, that the current study did not directly assess enzyme kinetics or perform 16S rRNA sequencing of the microbiota. Therefore, these proposed mechanisms remain hypothetical and are supported only by indirect metabolic profiling evidence.

It is crucial to clarify whether Borneol-induced metabolic shifts stem from the direct modulation of metabolic pathways or simply from increased substrate availability due to enhanced intestinal absorption. While the semi-quantitative increase in certain metabolites suggests a potential shift in metabolic flux, it is equally possible that the higher systemic concentration of parent compounds provides more substrate for basal enzymatic activity. Current data remain correlative, and the proposed modulation of CYP450 enzymes or gut microbiota should be considered hypothetical until direct enzymatic or 16S rRNA sequencing evidence is obtained.

### 4.4. Structural Validation of Multi-Target Neuroprotection

To bridge the gap between chemical identification and pharmacological efficacy, we integrated network pharmacology and molecular docking. Our analysis revealed that the eight core brain-penetrating exogenous metabolites are significantly enriched in the Arachidonic acid metabolism and TNF signaling pathways, which are central to post-ischemic inflammation and neuronal death.

Crucially, topological analysis identified PTGS2 (COX-2), TNF-α, and AKT1 as the primary hub targets. Subsequent molecular docking provided computational support that the synergistically enriched metabolites bind robustly to these targets. The exceptional spatial fit and high binding affinities of Coptisine with TNF-α (−9.2 kcal/mol) and Oxyresveratrol with PTGS2 (−8.3 kcal/mol) substantiate their direct role in neutralizing the neuroinflammatory storm. Simultaneously, the interaction of Gomisin H with AKT1 supports the activation of neuronal survival signaling. Furthermore, Borneol co-administration upregulated essential endogenous neuromodulators like Inosine and Pantothenate (Vitamin B5) [32], implying a holistic restoration of the brain’s energetic and osmotic homeostasis.

### 4.5. Limitations and Future Perspectives

Despite these promising findings, several critical limitations must be acknowledged. First, the characterization of metabolites relied primarily on qualitative high-resolution MS/MS matching; absolute quantification of their concentrations in the CSF was not performed due to the current lack of commercially available standards for several novel rearranged metabolites. Future studies employing targeted LC-MS/MS are necessary to determine whether these metabolites reach meaningful therapeutic thresholds in the brain.

Second, the relatively small sample size (n = 4–5 per group), necessitated by the technical complexity of rodent CSF extraction, may limit the statistical power of the multivariate analysis. While permutation testing was performed to exclude overfitting in the OPLS-DA models, research with larger cohorts is warranted to confirm the robustness of these metabolic shifts. Third, while CSF detection is a widely accepted proxy for central exposure, it cannot precisely distinguish the anatomical route of entry (e.g., endothelial BBB vs. epithelial BCSFB) or provide the high spatio-temporal resolution required to map parenchymal distribution.

Furthermore, while we predicted precise metabolic transformations and demonstrated high-affinity binding in silico, the exact enzymatic drivers (e.g., specific CYP450 isoforms) and the functional verification of BBB transport kinetics require further investigation using in vitro enzymatic assays and in vivo pharmacokinetic validation. Notably, our findings regarding the role of Borneol in modulating metabolic enzymes or gut microbiota remain hypothetical. Future work involving microbiome-depleted models and targeted enzyme inhibition is essential to confirm these complex interactions. Finally, the molecular docking results, though benchmarked against known inhibitors like SPD304, are predictive in nature. Because they lack a dynamic biological context, rigorous biochemical assays such as Surface Plasmon Resonance (SPR) or Western blot are needed to definitively confirm these direct target engagements and their downstream signaling effects.

## 5. Conclusions

In summary, this study established a systematic strategy integrating UHPLC-Q-TOF-MS/MS, metabolomics, network pharmacology, and molecular docking to decipher the in vivo fate and therapeutic mechanisms of dragon’s blood resin for ischemic stroke.

By comparatively analyzing plasma and cerebrospinal fluid (CSF) profiles, we elucidated the CNS-penetrating material basis of dragon’s blood. Our findings suggested a distinctive “pro-drug” mechanism driven by peripheral biotransformation, where macromolecular precursors underwent profound convergent metabolism—specifically through deglycosylation, demethylation, and hydroxylation—to yield potent, BBB-permeable active effectors such as Oxyresveratrol in the brain.

Crucially, this study provides novel structural and metabolic insights into the traditional role of Borneol as a “guide drug.” Co-administration with Borneol not only physically enhanced the CNS exposure of lipophilic flavonoids and lignans (e.g., Gomisin H) but also significantly modulated the systemic metabolic flux, which we hypothesize may involve enzyme or microbiota modulation, potentially generating unique, highly bioactive metabolites like Coptisine within the CSF. Subsequent computational validations suggested that these Borneol-enriched effectors exhibit strong predicted binding affinities with central hub targets (TNF-α and PTGS2), potentially suppressing the neuroinflammatory cascade while restoring the energetic microenvironment (e.g., Inosine, Pantothenate) of the ischemic brain.

These integrated findings substantiate the multi-component, multi-target pharmacological foundation of dragon’s blood and offer robust scientific evidence for the dual synergistic mechanism of Borneol as a vital adjuvant in the clinical management of central nervous system diseases. Future research will focus on clarifying these proposed mechanisms through 16S sequencing and enzymatic validation to further refine our understanding of Borneol’s role as a metabolic modulator.

## Figures and Tables

**Table 1:** The botanical characteristics, geographic distribution, and ethnomedicinal applications of *Dracaena cochinchinensis*.

Region (Country/Province)	Local Name	Ethnomedicinal Use	Plant Parts Used	References
China (Yunnan/Guangxi)	Xuejie	Promote blood circulation, heal wounds, treat stroke	Stem resin	[1]
Vietnam	Huyết Giác	Invigorate blood, treat rheumatism and pain	Resin/Stem	[2]
Laos	Chan Daeng	Treatment of fractures, contusions, and ischemia	Resin	[3]
Cambodia	Angraè daèk Tiên Ngôm	Antiseptic, stopping bleeding, stroke recovery	Roots/Stem	[4]
Thailand	Jun-Daeng/Chan Daeng/Nam Ton	Tonic, blood purifier, anti-inflammatory	Resin/Wood	[5]
Indonesian and Malaysian Peninsula	jernang/Darah Naga	Strong healing activity, treating gastric ulcers or intestinal-related cancers	Resin	[6]

**Table 2:** Summary of representative prototypes and their metabolic fates.

Prototype Name	Metabolite Name	Metabolic Pathway	Class	Plasma (DB)	Plasma (DB + B)	CSF (DB)	CSF (DB + B)
Betaine	Betaine	Prototype absorption	Amino acid	+	+	+	+
Gomisin H	Gomisin H	Prototype absorption	Lignan	+	+	+	+
Isorhapontigenin	Oxyresveratrol	Demethylation	Stilbene	−	+	+	+
Mulberroside A	Oxyresveratrol	Deglycosylation	Stilbene	−	+	+	+
Resveratrol	Oxyresveratrol	Hydroxylation	Stilbene	+	+	+	+
Berberrubine	Coptisine	Cyclization	Alkaloid	−	−	−	+
Dactylin	Cyanidin-3-glucoside	Deglycosylation	Flavonoid	−	+	+	+
Nobiletin	Artemitin	Demethylation	Flavonoid	+	+	−	−
Palmitoleic acid	Oleic acid	Chain elongation	Fatty acid	+	+	−	−

Note: “+” indicates detected; “−” indicates not detected.

**Table 3:** Molecular docking results of core CSF metabolites with hub targets.

Ligand	Hub Target	PDB ID	Vina Score (kcal/mol)	Cavity Volume (Å^3^)	Key Interacting Residues
Coptisine	TNF-α	2AZ5	−9.2	1263	ARG-82, TYR-87
Oxyresveratrol	PTGS2 (COX-2)	5IKQ	−8.3	10317	CYS-36, CYS-47
Gomisin H	AKT1	4EJN	−6.6	9015	GLN-59, VAL-270

## Data Availability

The original mass spectrometry data presented in the study are included in the article/Supplementary Materials; further inquiries can be directed to the corresponding author.

## Supplementary Materials

The following supporting information can be downloaded at https://www.mdpi.com/article/10.3390/metabo16050327/s1: Figure S1: 200-iteration permutation test for the OPLS-DA model; Figure S2: Molecular docking of the positive control SPD304 with TNF-α (PDB ID: 2AZ5); Table S1: Identification of chemical constituents in Dragon’s Blood resin by UHPLC-Q-TOF-MS/MS; Table S2: Identification of dragon’s blood-derived prototypes and their in vivo metabolites in rat plasma and cerebrospinal fluid (CSF).
