# Supplementary material for: Unveiling the Brain-Penetrating Material Basis of Dragon’s Blood: Identification of Active Metabolites and Metabolic Pathways for Ischemic Stroke Therapy
Source: Metabolites. 2026 May 14;16(5):327. doi: 10.3390/metabo16050327 (PMC13209074; doi:10.3390/metabo16050327)
Supplement: Supplementary file 1 [file metabolites-16-00327-s001.zip › Supplementary Materials-fig S1&S2.pdf]

Supplementary Materials

Figure S1. 200-iteration permutation test for the OPLS-DA model.

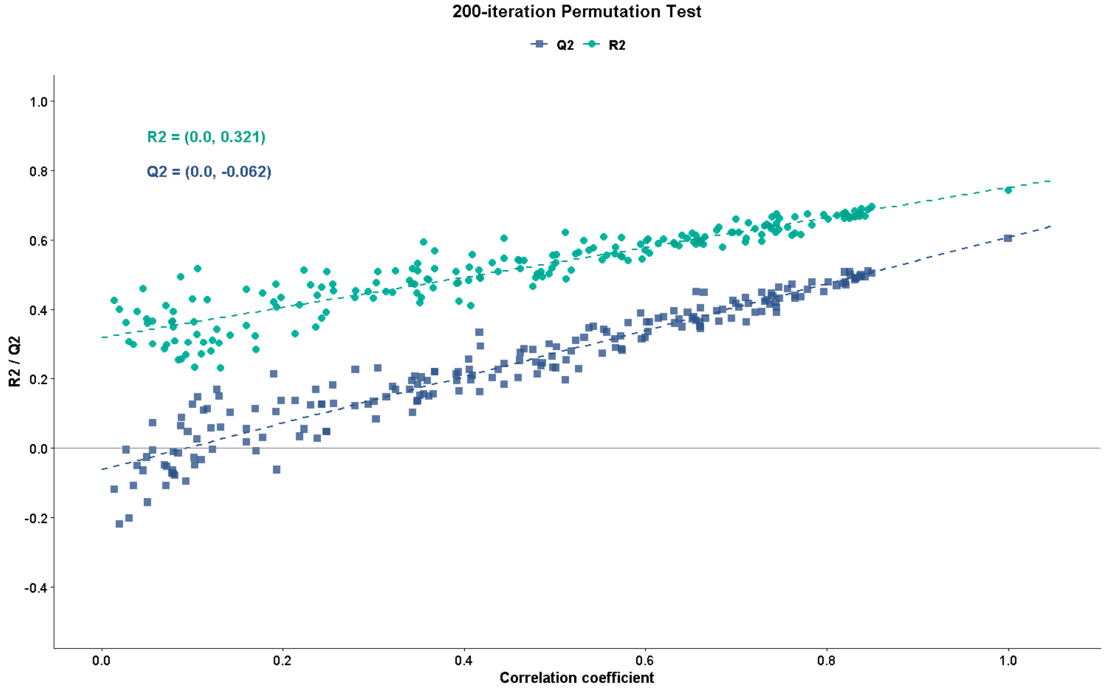

The intercepts of R2 and Q2 indicate that the model is statistically reliable and lacks significant overfitting.

Figure S2. Molecular docking of the positive control SPD304 with TNF-α (PDB ID: 2AZ5).

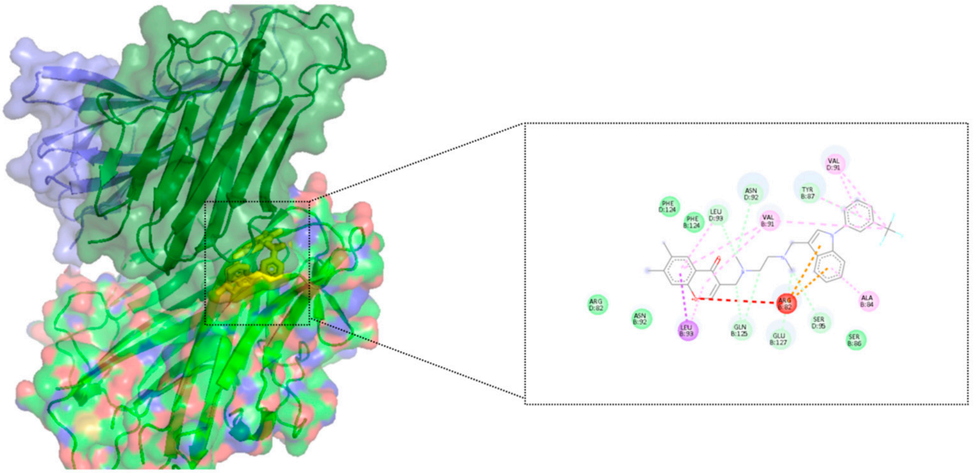

SPD304 is shown docked into the dimeric interface of TNF-α, demonstrating its predicted binding orientation and interaction residues.

Target Protein	PDB ID	Ligand	Vina Score (kcal/mol)	Role

Target Protein	PDB ID	Ligand	Vina Score (kcal/mol)	Role
TNF-α	2AZ5	SPD304	-9.6	Positive Control
TNF-α	2AZ5	Coptisine	-9.2	Key Metabolite
